# Everybody's Page

**Published:** 1889-11-23

**Authors:** 


### Everybody's Page.

The exposure of the eye to intense light has often caused total blindness.

Lock-jaw or tetanus is said to be amongst the diseases of which the "germ" has been discovered. In certain districts, where fish refuse is found, tetanus is very common, and Brieger believes that the tetanus-germ finds a congenial soil in such refuse in which to flourish.

It is not generally known that a decoction of tobacco sometimes relieves the agony of gout. A great sufferer says he finds it more efficacious than anything else. Four ounces of tobacco are infused in a pint of boiling water, and flannels wrung out in this infusion are swathed round the afflicted limb. It sounds a little extiavagant perhaps to smokers.

The difficulties which the authorities at Assouan have to contend with in their efforts to keep down smallpox do not arise from any objection to vaccination on the part of the Arabs, who submit readily to the operation, thinking the marks rather decorative no doubt, but from the difficulty of getting those people vaccinated who claim the protection of the different European consulates.

The harmless Tarantula spider was formerly credited with originating a singular epidemic of hysteria or chorea, which once took place in the town and neighbourhood of Tarentum, in Italy. Anyone bitten by the spider was affected with the disease, which was called tarantismus, and exhibited itself either in profound melancholy or a continuous convulsive movement of the body. Music and dancing were the treatments the patients underwent, and some allege this to have been the origin of the dance the "Tarantella."

Some time ago Dr. Hammond, of New York, experimented on himself with cocaine, fancying that it aided the mental and moral tone, whereas alcohol did the opposite. He found that three grains produced a great disposition to talk, with vivid imagination. Writing was accomplished with great ease, and wonderful progress was made with a medical work which he was preparing. On the following morning he found the work to be composed of incoherent sentences and disconnected ideas, being in fact utterly nonsensical. Since then his opinion of the powers of cocaine have changed.

Many a convalescent who seemed on a fair road to health has had a serious relapse after what seemed a harmless meal of fish. This has generally been found to be owing to bad cooking; the fish itself is wholesome enough if properly steamed, but if slowly boiled and served in a sodden condition, or if fried in fat over a black fire, the eating of it is likely to be followed by evil consequences. Our health depends quite as much on the way our food is cooked as on the food itself, therefore it is a pity when women of all grades refuse to avail themselves of the excellent cooking schools now to be found in every large town.

Jungle fever, Terai fever, Bengal fever, etc., of which we hear so much from Anglo-Indians, are all merely exaggerated forms of the common intermittent fever, or ague. In remittent fevers, however, there is not the distinct intermission between the hot and cold stage of ague, and the sufferings are more severe. Very often the patient becomes delirious, the eyes are bloodshot, and the whole stage of the paroxysm, which usually lasts about twelve hours, is very trying to the nurse and very weakening to the patient. Unluckily, when the victim returns to England, the fever frequently comes with him, and for some years he may feel the effects of his Indian experience.

The immunity from smallpox of vaccinated and revaccinated nurses in smallpox hospitals is valuable testimony to the efficacy of vaccination.

Whether the poor girl at Stockport died from the effects of antipyrin or not, her death is a warning to those misguided persons who will doctor themselves with new or little known remedies.

After the removal of dust from the eye, if pain and inflammation are still felt, a drop of castor oil should be placed in the eye with the feather end of a quill, and a bandage worn for a few hours to secure rest and exclusion of light.

Lemonade and orangeade are now well known as cooling drinks for invalids, but apple water is seldom used, though quite as refreshing. It should be made with cooking apples rather sour and very juicy; peel two, cut them in slices, put them in a jug with two ounces of sugar, pour on a quart of boiling water. When quite cold, strain and serve.

During an epidemic the fearful often make the mistake of utterly changing their mode of life. In a cholera epidemic, for instance, some will refuse all vegetables and take great quantities of alcohol; the change affects their constitution, they get out of condition, and are likely to fall victims to the fatal scourge. It is far wiser to pursue the "even tenor of our way," living moderately and fearing nothing.

Dr. G. Descourtis has been waging war against what he considers the over education of young girls; he says woman's *role* is forgotten in modern society. The future of sons and soldiers all depends on the health of their mothers, and yet in the struggle for a young girl to carry a diploma in her pocket, anæmia and tuberculosis are forgotten. "Strength," says the doctor, "is a man's attribute; grace, that of woman, but grace does not exclude health, and health is only attained by a healthy life and exercise." Ambitious parents, take warning!

Willam Salmon, Professor of Physick at the Ditch-side, Holbourn Bridge, 1693, is very learned about the preparations made from man's skull. He advocates the use of a skull which has been buried against one which is fresh and green against the epilepsie. "In a certain patient we had given more than thirty doses of skulls not buried without any success at all, whereas upon the exhibition of six or seven doses of that which had been long buried, the same patient miraculously recovered, so as the disease never returned any more." One feels much obliged to doctors for not using skulls nowadays; it savours of South Sea Islanders.

The evil habit of going too long without food is one from which many people suffer in the present hurrying age. Men sit in their offices, women rush about at their shopping, and both become so absorbed in their interests that the period of hunger is allowed to pass, and the period of fatigue and depression to set in. The worst of it is that once the second stage is reached, the desire for food is gone, and after many hours' abstinence the man or woman is too exhausted to digest a meal when they get it. To avoid this extreme, it is only necessary to take the most light and rapid repast during the hungry stage. A glass of milk, a cup of bovril, or merely a biscuit while hungry will prevent the after loss of appetite. And yet many prefer to ruin their health rather than take the trouble to turn into a dairy shop and drink a glass of milk.

